# Tracking the allocation of attention using human pupillary oscillations

**DOI:** 10.3389/fpsyg.2013.00919

**Published:** 2013-12-10

**Authors:** Marnix Naber, George A. Alvarez, Ken Nakayama

**Affiliations:** ^1^Vision Sciences Laboratory, Department of Psychology, Harvard UniversityCambridge, MA, USA; ^2^Social and Behavioural Sciences, Cognitive Psychology Unit, Leiden UniversityLeiden, Netherlands

**Keywords:** pupil, oscillations, frequency tagging, attention, SSVEP, attentional blink, PFT, tracking

## Abstract

The muscles that control the pupil are richly innervated by the autonomic nervous system. While there are central pathways that drive pupil dilations in relation to arousal, there is no anatomical evidence that cortical centers involved with visual selective attention innervate the pupil. In this study, we show that such connections must exist. Specifically, we demonstrate a novel Pupil Frequency Tagging (PFT) method, where oscillatory changes in stimulus brightness over time are mirrored by pupil constrictions and dilations. We find that the luminance–induced pupil oscillations are enhanced when covert attention is directed to the flicker stimulus and when targets are correctly detected in an attentional tracking task. These results suggest that the amplitudes of pupil responses closely follow the allocation of focal visual attention and the encoding of stimuli. PFT provides a new opportunity to study top–down visual attention itself as well as identifying the pathways and mechanisms that support this unexpected phenomenon.

## Introduction

Paying attention to items and events outside ones central gaze is a key cognitive skill (James, 1890; Posner, 1980). For instance, a driver's main focus is the road, but attention may need to be diverted to the pedestrians on the sidewalk as well. Visual attention is the cognitive process of (pre)allocating mental resources to particular locations, features, or objects in a visual scene (e.g., Scholl, 2001; Naber et al., 2011) to improve sensory processing of the selected information (Corbetta et al., 1990; Motter, 1993; Desimone and Duncan, 1995; Hillyard et al., 1998; Roelfsema et al., 1998; Somers et al., 1999; Kastner and Ungerleider, 2000; Treue, 2001; Silver et al., 2007). However, observers cannot attend to everything in their surroundings at the same time because the visual system has serious limitations in processing capacity (Broadbent, 1958; Neisser, 1967; Schneider and Shiffrin, 1977; Tsotsos, 1990; Verghese and Pelli, 1992). Therefore, attention needs to be divided between many competing features, some of which automatically attract more resources than others (e.g., Treisman, 1969; Eriksen and Eriksen, 1974; Duncan, 1984). Hence, there can be parts of the visual scene that receive focused attention and parts that receive none or fewer attentional resources. The perception of the latter is extremely limited (Rensink et al., 1997; Mack and Rock, 1998; Most et al., 2005; Cohen et al., 2011) and consequently attentional slips sometimes lead to undesirable events such as accidents (Reason, 1990). As attentional competition and capacity limitations can have serious repercussions for everyday life, it is important to investigate their underlying mechanisms.

Visual attention is usually measured by assessing performance outcomes on a task. In a typical experiment, observers are cued to attend a particular object, which leads to faster and more accurate report of its properties as compared to unattended objects (Averbach and Coriell, 1961; Eriksen and Hoffman, 1972; Posner, 1980; Nakayama and Mackeben, 1989). The deployment of attention is, however, considerably variable over time (e.g., Martínez et al., 2001) and it has been a challenge for researchers to measure its deployment throughout a single experimental trial (Bennett and Pratt, 2001; Tse et al., 2003). To successfully relate small and short-term changes in attention to behavior, we need to be able to measure its dynamics *on-line*. Here we present a novel *pupillometric* method that serves as a tool to measure attention over time and to predict behavioral performance on a trial-by-trial basis.

We demonstrate that attention enhances not only performance on a task, but also pupil responses. We employ a method similar to steady-state visual evoked potentials (SSVEP) used in MEG/EEG studies (Regan, 1989; Morgan et al., 1996; Müller et al., 2003; Störmer et al., 2013). However, rather than using electrophysiological signals to track the dynamics of attention, we use frequency tagged pupillary responses. Specifically, we induce pupil oscillations by modulating luminance levels of target objects and distractor objects at different frequencies, and show that the amplitude of these pupil oscillations track focal attention allocated to a specific flickering object. We have termed this novel attentional tracking method Pupil Frequency Tagging (PFT) and demonstrate its application and potential in three experiments.

## Experiment 1

The PFT method requires repetitive oscillations in the brightness of stimuli (dark-light-dark-light…), in combination with continuous measures of pupil diameter using an eye-tracker. If a stimulus is relatively brighter than its background, then its appearance will trigger pupil constriction and its disappearance will trigger pupil dilation. Our question was simple: Would the amplitudes of these pupil responses be modulated by attention? Before measuring possible effects of attention, we first determined the highest frequencies where satisfactory pupil responses could be obtained by presenting a full-screen flickering stimulus.

### Materials and methods

#### Observers

Thirteen students participated in Experiment 1. All participants had normal or corrected-to-normal vision, were naïve to the purpose of the experiment, and gave informed written consent before the experiment. The experiments conformed to the ethical principles of the Declaration of Helsinki and were approved by the local ethics commission of Harvard.

#### Stimuli and apparatus

**Figure 1:**
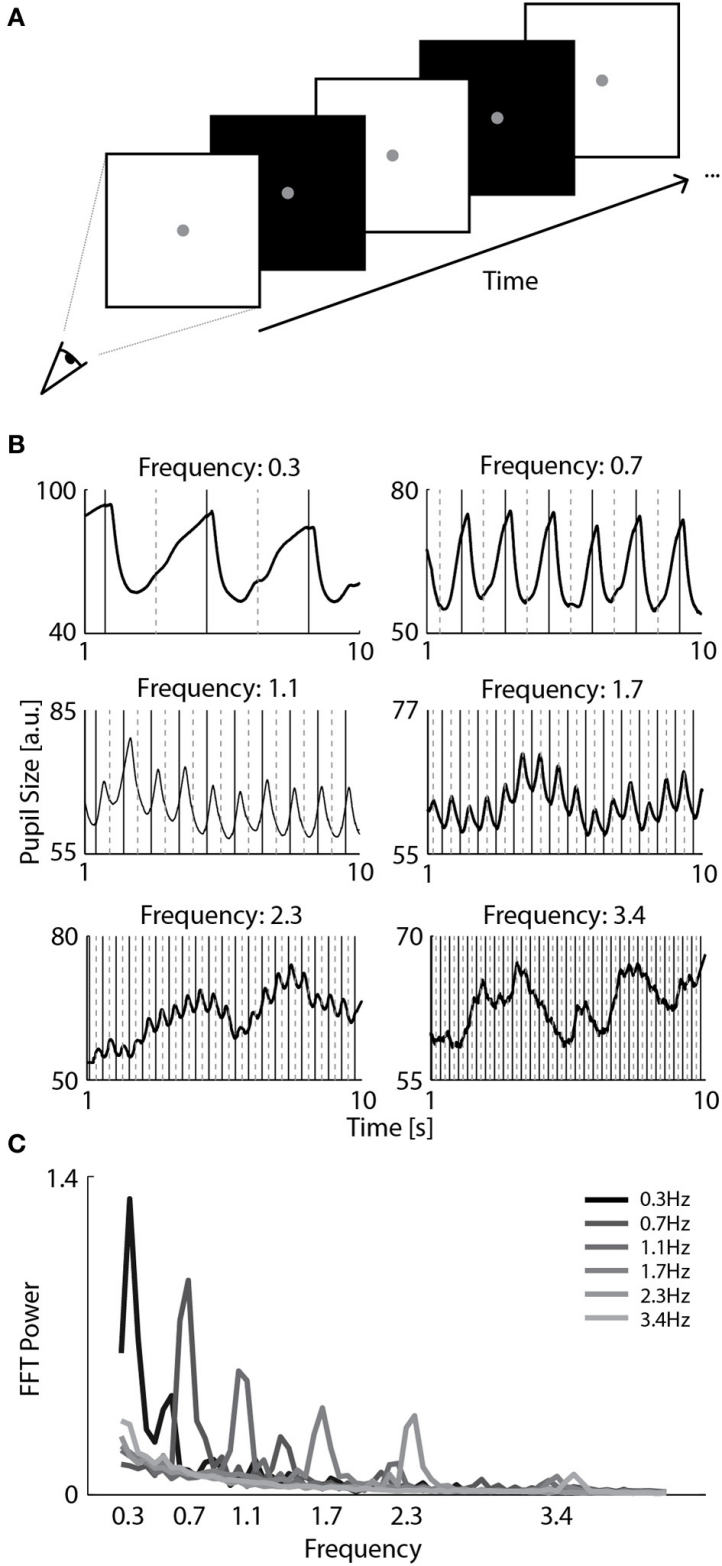
**Pupillary responses to a range of screen flicker rates. (A)** Observers viewed full monitor screens that flickered at a particular frequency rate (0.3, 0.7, 1.0, 1.7, 2.3, or 3.4 Hz) while their pupil size was recorded with a camera. **(B)** Examples of pupil size of a selected observer as a function of time in six separate trials with distinct flicker frequencies. The solid and dashed vertical lines indicate the onsets of white and black screens, respectively. **(C)** Average spectrum of FFT power per flicker frequency across all observers.

To measure the effects of changes in perceived brightness on pupil size, observers viewed a blank screen that flickered at either 0.3, 0.7, 1.0, 1.7, 2.3, or 3.4 Hz (Figure 1A). The monitor screen was 30 by 24 in visual degrees and the fixation point was 0.25° in diameter. The screen, fixation, and backgrounds were either black (1.65 cd/m^2^), gray (16.46 cd/m^2^), or white (61.10 cd/m^2^).

Stimuli were presented on a 21″ CRT screen at a fixed viewing distance of 70cm. Observers' heads were supported by a chin- and forehead-rest. The resolution and refresh rate of the screen was 1600 × 1200 pixels and 85 Hz. Observer's pupil size of one eye was tracked with an infrared sensitive camera at a rate of 1000 Hz.

#### Procedure

Observers viewed a full screen that alternated between black and white at a specific frequency while their pupil size was recorded. Observers were instructed to fixate at the center dot but pay close attention to the flicker rates. A different screen alternation frequency was randomly selected per trial (2 trials per frequency). Observers could take breaks between trials and start each trial by pressing a button. The experiment consisted of 12 trials of 10 s each.

#### Analysis

The strength of pupil oscillations was analyzed by conducting a Fast Fourier Transform (FFT) that produces a power spectrum across frequencies. The EyeLink pupil tracking system outputs pupil size in arbitrary units that depend on variable factors such as the camera's pupil detection parameters and the observer's viewing distance to the screen. Nonetheless, we could roughly estimate that a pupil size unit of 100 corresponded to a pupil diameter of approximately 6 mm and a unit of 40 to 3 mm (see Figure 1B). Pupil size and gaze location was interpolated with a cubic spline fit during blinks. Pupil size recorded in the first second of each trial was removed from analysis to control for confounding effects on pupil size due to transient onset responses and because observers needed some time to become oriented after trial onset.

### Results and discussion

In Experiment 1, observers viewed a full-screen flickering stimulus, where the flicker frequency varied across trials (0.3, 0.7, 1.0, 1.7, 2.3, and 3.4 Hz; Figure 1A). As shown by the continuous changes in pupil size synchronous to the flicker rate of the stimulus in Figure 1B, most flicker frequencies induced consistent pupillary oscillations. Next, we determined whether a FFT frequency spectrum analysis on the pupil oscillations accurately which frequency was presented on each trial. As shown in Figure 1C, the power magnitudes in the FFT frequency spectrum were selectively enhanced for the presented flicker frequencies. The power of each present frequency was significantly larger than the power of absent frequencies across observers [*t*_(12)_ >= 2.73, *p* <= 0.018; for all statistical comparisons, see Table A1]. The peak in power of the highest flicker frequency (3.4 Hz) was also discernibly higher than other frequencies on most trials, except for 3 out of 13 observers whose pupillary responses were too noisy to get reliable magnitudes at that frequency. Hence, we conclude that flicker frequencies up to 2.3 Hz induce consistent, measurable pupillary oscillations in all observers. In the following experiment, we use this frequency to investigate whether we can measure attentional effects on pupil responses at a relatively high temporal resolution.

## Experiment 2

Having established that an FFT spectrum analysis of pupil oscillations accurately indicates visual flicker frequencies up to ~2.5 Hz, we investigated whether attention modulates oscillations amplitudes. To do so, we presented four separate stimuli with distinct locations and flicker frequencies to observers while recording pupil responses as a function of attended location. The idea was that each flicker frequency left its own oscillatory trace in the pupil and that the strength of this oscillation can be measured by determining the peak power in the FFT spectrum analysis.

### Materials and methods

#### Participants

A new group of 15 students participated in Experiment 2. All other aspects were similar to Experiment 1.

#### Stimuli and apparatus

**Figure 2:**
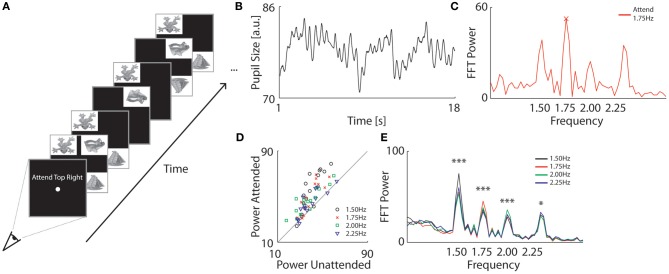
**Pupillary assessment of spatial focal attention. (A)** Procedure of Experiment 2. Observers fixated at the screen center and counted the number of times the attended object flipped upside down. Each quadrant had an object within a white rectangle that flickered off-and-on at a separate frequency (1.5, 1.75, 2.0, and 2.25 Hz). **(B)** Example of an observer's pupil size during a single trial (*black*). The four flickering stimuli induced continuous pupil oscillations. **(C)** Example of a single trial FFT power spectrum analysis of the pupil trace in **(B)**. The four peaks at the presented frequency indicate that each quadrant left an oscillatory trace in the pupil. In this trial, the observer specifically attended an object that flickered at 1.75 Hz. This frequency was the strongest represented oscillation in the pupil trace. **(D)** FFT Power values for attended frequencies as a function of power averaged across the unattended frequencies. Each data point represents the average power for an individual observer at a particular target frequency (see colored markers). Attended frequencies reliably induced enhanced pupil oscillations amplitudes as compared to unattended frequencies across observers. **(E)** Average FFT power spectrum analysis across observers as a function of attended frequency (see colors). Attended frequencies induced significantly higher power values than unattended frequencies (^*^p < 0.05, ^***^p < 0.001).

**Figure A1:**
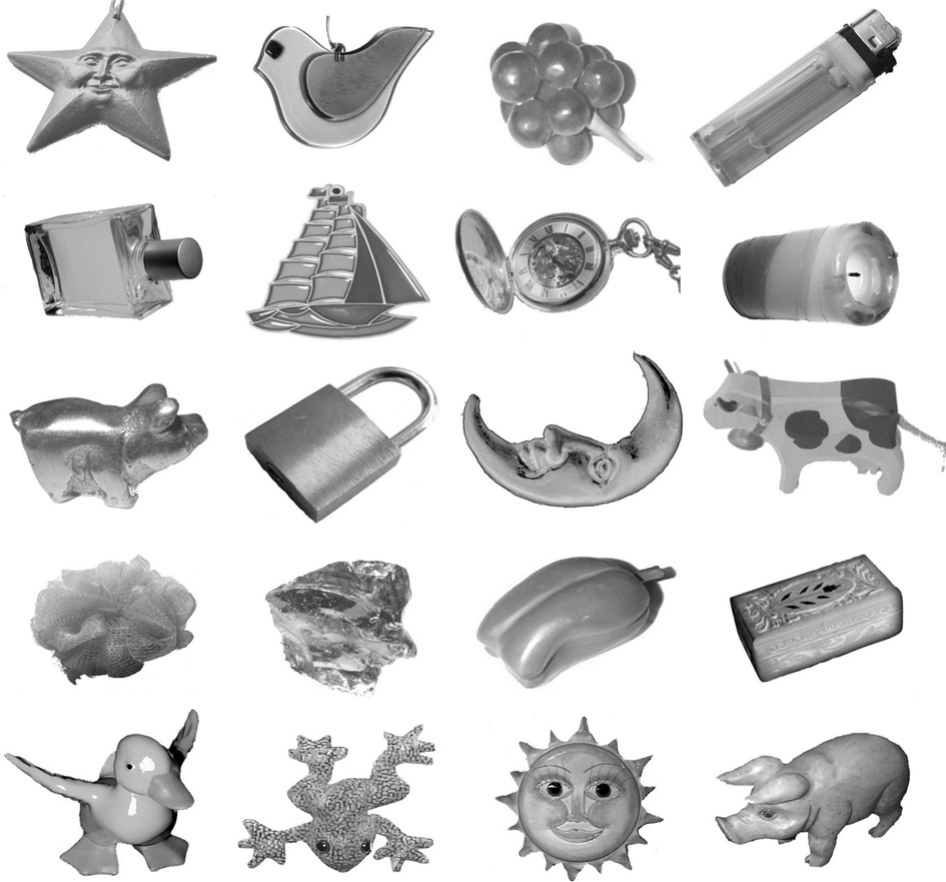
**Image set used in Experiment 2.** Objects were equal in size, luminance, and global contrast. Four images were randomly selected from this set per trial. Objects were collected from http://www.freeimages.co.uk/.

Observers viewed four stimuli that flickered at distinct frequencies (Figure 2A). Stimuli consisted of objects embedded in white quadrants that flickered on a black background at a frequency of 1.5, 1.75, 2.0, or 2.25 Hz. Each quadrant flickered at a distinct frequency, counter-balanced across trials. In addition, each of the objects was randomly flipped 3–6 times per trial for task purposes (see Procedure). The image set consisted of 20 separate objects adapted from http://www.freeimages.co.uk/ (Figure A1) and images were equalized in luminance (46.53 ± 0.34cd/m^2^) and contrast (19.71 ± 0.33cd/m^2^). The luminance and contrast was calculated by taking the average and standard deviation of the luminance values across all pixels, respectively. The objects in the images were also equalized in size (61.32% ± 0.01 of the total white rectangular image). All images were 11 by 8° in width and height, and were placed at the corners of the screen. The center and corner of the images were located 12.4 and 5.7° from the fixation dot, respectively.

#### Procedure

Observers were cued at the start of each trial to attend one of the four objects (Figure 2A). To ensure that observers were attending the cued object, they were instructed to count how many times it flipped upside-down. Specifically, all the objects flipped upside-down at random moments between appearances and disappearances during a trial, and the observer had to count these events specifically for the attended object. Which stimulus to attend was counter–balanced across trials. Experiment 2 consisted of 16 trials of 18 s each.

#### Data normalization

**Figure A2:**
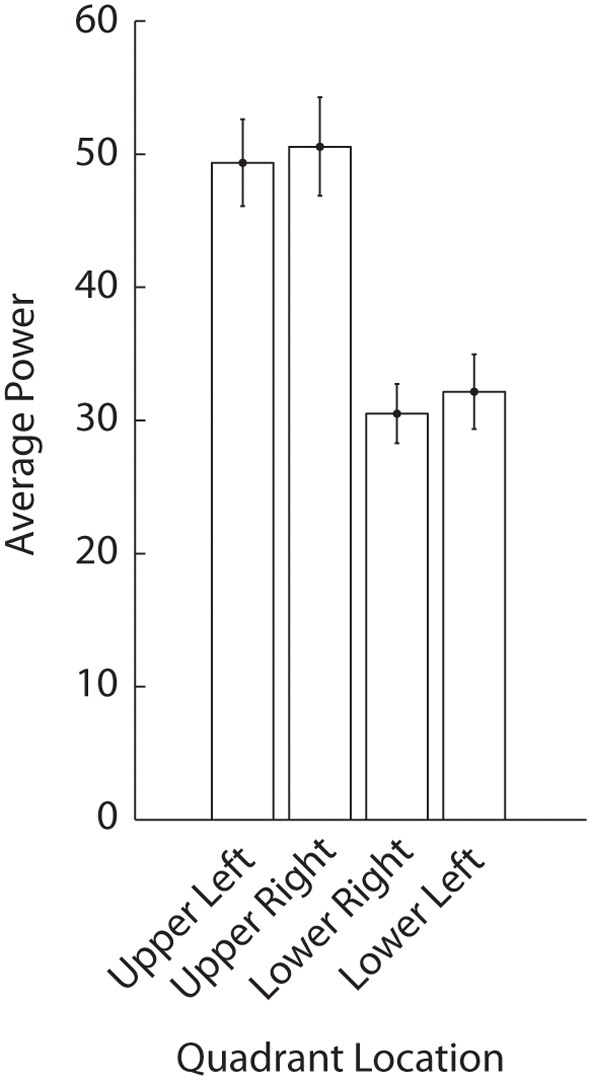
**Average pupil oscillation power per quadrant location in Experiment 2.** The flickering quadrant in the upper visual field induced the strongest pupillary responses.

Trials in which an observers' average gaze was more than one degree away from fixation were removed from the analysis (one trial on average per observer). To remove low frequency artifacts in pupil size due to overall arousal, pupil size traces were de-trended by subtracting low-pass filtered traces from the original data. The low-pass filter was a smoothing filter with a window size of two periods of the lowest flicker frequency (i.e., 2/1.5 Hz = 1.33 s). As flicker frequencies in the upper visual field tended to increase pupil oscillation amplitudes as compared to flicker in the lower visual field (see Figure A2), we controlled for visual field anisotropies and normalized each power value per location and per trial by subtracting the average power for that location across all trials (normalized power would show no differences in Figure A2). Similarly, lower frequencies also tended to induce slightly larger amplitudes in the FFT analysis (see Figure 1C) and we corrected this by subtracting the average power per frequency across all trials.

#### Analysis

We performed a trial-by-trial decoding of which stimulus was attended by examining the power at target and distractor frequencies on each trial. Decoding accuracy was calculated by computing the percentage of trials in which the peak power of the target frequency was higher than the peak power of all distractor frequencies.

### Results and discussion

Similar to Experiment 1, we first analyzed the oscillation frequencies in the pupil traces for each observer. As shown with an example trial in Figure 2B, the pupil traces consisted of ongoing oscillations with clear peaks and troughs. An FFT spectrum analysis depicted discrete power increases selectively at each presented flicker frequency (Figure 2C). This confirms that each stimulus' frequency left a separate trace in the pupil oscillations. For a yet unknown reason, the highest stimulus frequency of 2.25 Hz exhibited a slightly shifted peak to around 2.35 Hz. Otherwise, the pupil's dynamics closely reflected the flicker rates of the stimuli.

Of principal interest was whether the pupil oscillations could be altered by attention, and specifically whether the amplitude of pupil oscillations was selectively enhanced for the frequency of the attended object. To address this question, we first investigated whether the height of the peak in power in the FFT analysis was specifically increased for attended frequencies. Figure 2C shows the power spectrum of a single trial in which the observer attended the object that flickered at 1.75 Hz. In this particular example, the peak in power of the attended frequency was higher than the unattended frequencies. To see whether this effect of attention was consistent across trials and observers, we calculated the average power for the attended frequency (target) and unattended frequencies separately for each target frequency (averaged across trials) and for each observer. As shown in Figure 2D, most of the power values of the attended frequencies were higher than the unattended frequencies across all observers (see each marker) and conditions (see color). The average spectrum across observer per condition is depicted in Figure 2E and a repeated measures ANOVA confirmed that attention significantly enhanced pupil oscillations across observers at each frequency [main-effect of attention: *F*_(1, 14)_ = 19.74, *p* < 0.001; for *post-hoc* comparisons across conditions per frequency, see Table A2].

Next, we determined how well we could decode from the pupil power spectrum analysis which stimulus was attended *on any given trial*. The difference in pupil oscillation power between target and distracter frequencies was small (8 ± 5 units which corresponds to ~0.05 mm ± 0.03 mm) but strong enough (24 ± 13%) to correctly predict in ~3 of every 4 trials (i.e., 73 ± 20% of all trials) which location was focally attended.

In summary, flicker frequencies of attended locations were selectively facilitated in the pupil response amplitudes. A shift in focal attention to a target leaves an enhanced oscillatory trace in the pupil responses specifically at the target's flicker frequency. This enhancement was strong enough to accurately predict which location was attended per trial with fairly high accuracy. An increased level of focal spatial attention is thus assessable with the amplitudes of pupil oscillations as responses to the onset and offset of flickering stimuli. As such, the frequency tagging of stimuli and simultaneous measurement of pupil responses is a suitable on-line measure of the attentional focus.

## Experiment 3

In the previous experiment, pupillary responses to light revealed its strong dependency on sustained attention. In this section, we ask whether pupillary responses can track more dynamic aspects of attention as it fluctuates over the course of the trial.

### Materials and methods

Another separate group of 25 observers participated in Experiment 3.

#### Stimuli and apparatus

**Figure 3:**
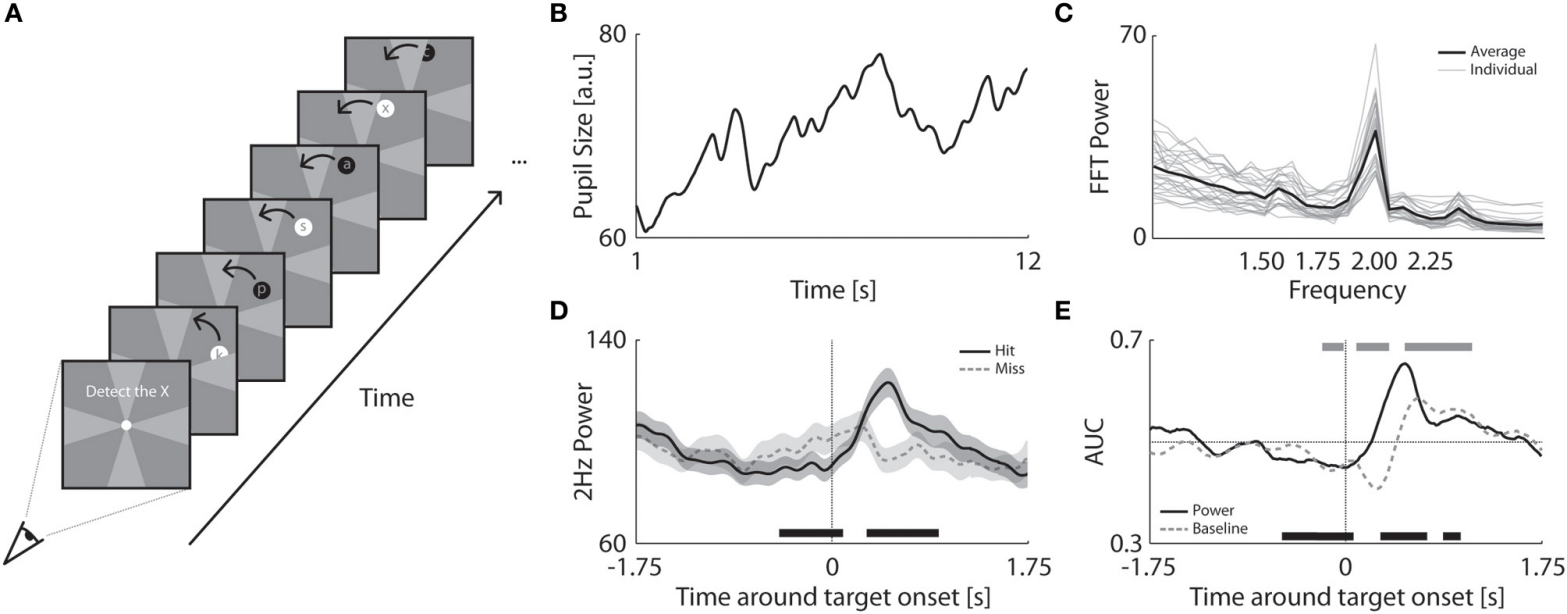
**Pupillary prediction of attentional resources and behavioral performance. (A)** Procedure of Experiment 3 in which a flickering disk (2 Hz) with a superimposed letter stream circled around fixation. Observers had to detect the “*x*” while fixating at the center dot. The disk moved behind occluders at the vertical and horizontal meridians. The occluders had the same color as the background but are here indicated in a brighter gray for clarification. **(B)** Example of an observer's pupil size trace in a single trial. **(C)** FFT power analysis per observer (*gray*) and averaged across observers (*black*). **(D)** Power at 2 Hz as a function of time around hit (*black*) and missed targets (*dashed gray*). The transparent patches around the average indicate the standard error. The patch at the bottom of the plot indicates at which time points the power between hit and missed targets significantly differed (*p* < 0.05). **(E)** Average AUC as a function of time around target onset for FFT power distributions (*black*) and baseline raw pupil size (*dashed gray*). Patches at the bottom and top indicate significantly higher or lower AUC (compared to 0.5) for power values (*black*) or pupil baseline (*gray*).

Observers tracked a flickering target that moved in the periphery (Figure 3A). The target stimulus consisted of a moving disk (4.65° diameter) that alternated between black and white at a fixed rate of 2 Hz. The disk moved 30° per second in a circular trajectory at a fixed 9.30° eccentricity from fixation. The disk moved for 12 s per trial, completing a full circle. Further, the disk was occluded and no targets were shown at the meridians (see gray wedges in Figure 3A) to ensure that the pupil amplitudes were not affected by anisotropies in visual detection sensitivities at the meridians (e.g., see Seiple et al., 2004). A stream of randomly changing alphabetical gray letters was superimposed on the disk and each letter change was in synchrony with the disk's 2 Hz alternation rate. The gray occluders at the meridians were 30 rotational degrees in width. To increase hardware performance for the display of smooth motion, the resolution and refresh rate of the screen was decreased to 1280 × 1024 and 60 Hz.

#### Procedure

Observers tracked the moving disk with a superimposed stream of changing letters. Observers were instructed to fixate but attend the letters and press a button every time the target letter “x” was presented. Two to four targets were shown per trial. The disk and stream of letters disappeared behind occluders around the meridian and no targets were shown when the disk was partially or fully occluded. The experiment contained 32 trials of 12 s each.

#### Data normalization

Similar to Experiment 2, pupil size traces were filtered to remove slow changes (e.g., across multiple trials) in pupil size due to arousal. Pupil size traces were, however, filtered with a less sensitive smoothing filter (i.e., a larger window size of 4 s for the subtracted low-pass filter) to preserve low frequency changes in pupil size within the time period of −2 to 2 s around target onset. In contrast to Experiment 2, power values were *not* normalized for target location because of the disk's dynamic location.

#### Analysis

**Figure A3:**
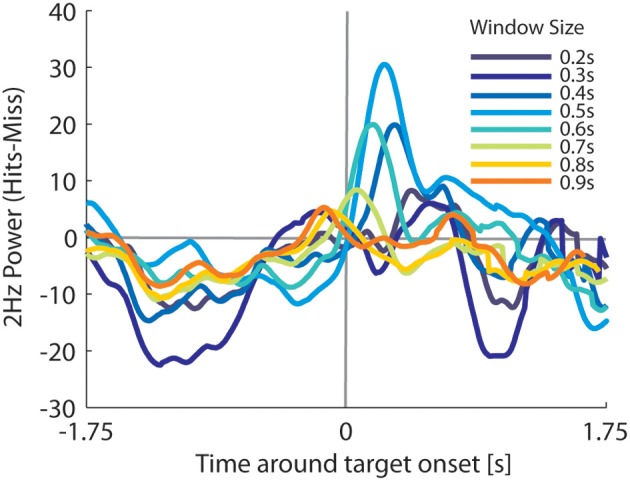
**Average difference in 2 Hz power between hits and misses as a function of time around target onset per size of the sliding window in the FFT power spectrum analysis**.

We analyzed the size of pupil oscillation amplitudes to determine whether they can be used to discern between trials when the target was detected (Hit trials), and trials when the target was missed (Miss trials). A FFT analysis was used to compute 2 Hz oscillation power as a function of time around target onset. Power values were extracted from pupil traces within a 0.5 s window (i.e., one frequency period of 2 Hz) that slid from −2 s to 2 s around target onset (for the effect of window size on oscillation power, see Figure A3). We then assessed whether these power values could be used to discriminate hits from misses using Signal Detection Theory (Green and Swets, 1966). That is, for different power thresholds, we classified trials above threshold as Hits, and measured the proportion of Hit trials correctly classified relative to the proportion of miss trials incorrectly classified. If Hits and misses are discernible in terms of their 2 Hz power, then for certain thresholds across the range of possible thresholds, there should be more Hit trials correctly classified than Miss trials incorrectly classified. Thus, by varying the power threshold across the full range of possible values determined separately for each observer, we can sweep out a classical receiver operating characteristic (ROC) curve (for details, see Green and Swets, 1966), plotting the proportion of incorrectly classified misses (i.e., misses exceeding this threshold) against the proportion of correctly classified hits (i.e., hits exceeding this threshold).

If power is identical between Hit trials and Miss trials at all 2 Hz power thresholds, then the ROC curve will be a straight line ranging from zero to 1.0. However, to the extent that hit trials and miss trials are discernible, the function will be curved (e.g., an upward curve would indicate a greater proportion of hit trials than miss trials across a range of power thresholds). The magnitude of curvature was determined by calculating the Area Under the Curve (AUC), with 0.5 being chance (50%) discrimination between hits and misses, and 0 or 1.0 (100%) being perfect discrimination between hits and misses. Thus, AUC serves as a measure of classifier accuracy that does not depend on the particular threshold used, since it summarizes across the full range of possible thresholds.

For comparison purposes, we also performed this same ROC analysis using raw pupil size.

### Results and discussion

Observers tracked a moving disk to detect target letters that were briefly presented on the disk (Figure 3A). As the area of the flickering disk was much smaller than the quadrants in Experiment 2, we first determined whether the disk could also induce coherent pupil oscillations (Figure 3B). The FFT analysis of individual pupil traces confirmed that the moving disk evoked strong 2 Hz oscillations in pupil size. Figure 3C shows the power of the FFT analysis at 2 Hz was significantly larger than the other frequencies across all observers [*t*_(24)_ = 17.52, *p* < 0.001]. Thus, despite its small size, peripheral location, and constant motion, the disk induced clear pupil responses at a rate of 2 Hz.

**Figure A4:**
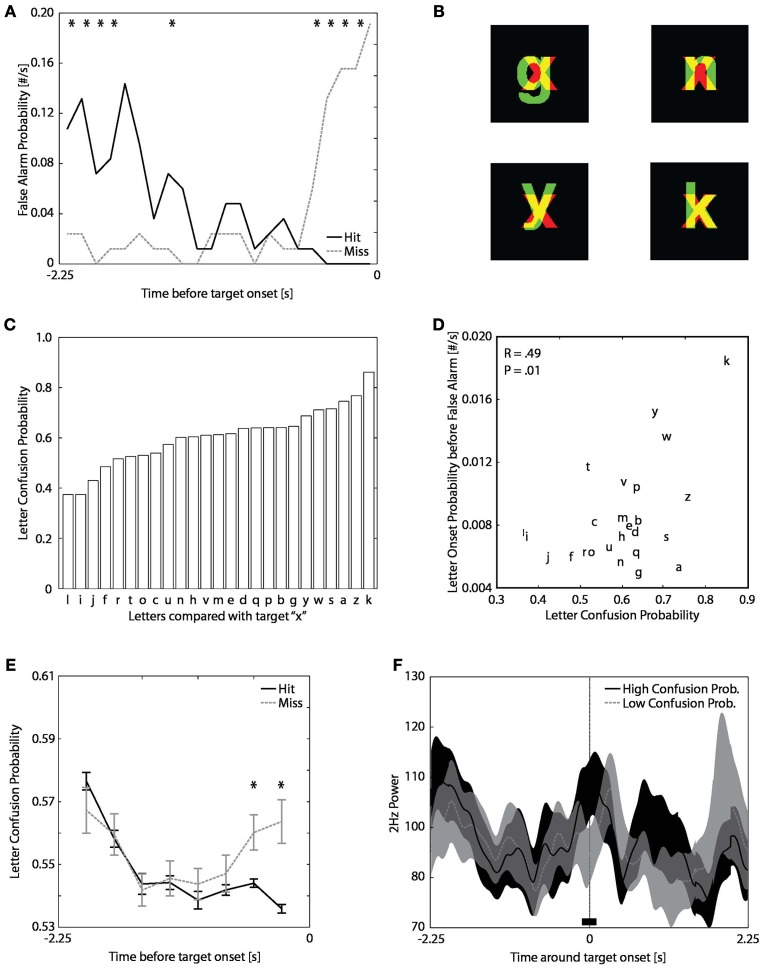
**False alarms, confusing letters, and misses. (A)** Probability that an observer false alarmed to the onset of a non-target as a function of time before target onset per hits (*black*) and misses (*gray*). (^*^p < 0.05) **(B)** Examples of overlapping letters. Images were created by presenting each of the two letters in the separate *red* and *green* color channel such that the overlap in letters is indicated in *yellow*. **(C)** The probability to confuse a non-target letter with the letter “x” based on the amount of overlap in **(B)**. **(D)** Letter onset probability before false alarm as a function of letter confusion probability per letter. The probabilities were based on the 4 preceding letters before the false alarm response (i.e., a one second window). **(E)** Letter confusion probability as a function of time before target onset per hits (*black*) and misses (*gray*). **(F)** Power at 2 Hz outputted by FFT analysis on pupil size around target onset per condition that the preceding 4 letters had an average high (*black*) or low (*gray*) confusion probability with the target letter “x”.

Target detection performance was high (78 ± 14% hits on average across observers), but a significant portion of the targets were missed. The question remains whether we could predict a successful target detection within a single trial based on pupil oscillations alone (2 Hz). In other words, could we determine the amount of attention allocated to the stimulus from the pupil oscillations and predict whether observers would detect or miss a particular target? To address this, we examined how the amplitude of pupil oscillations developed during correct target detection (i.e., hits) as compared to target misses. Specifically, we moved a sliding window of 0.5 s over the pupil traces and calculated the FFT power at 2 Hz per time point (for details, see Methods). For successfully detected targets, 2 Hz power decreased before target onset and then briefly increased after target onset (Figure 3D; see Figure A3 for the effects of the window size). The power showed an opposite pattern for missed targets, where it increased around target onset but did not reach a strong peak afterwards. These results suggest that pupil power *before* the actual target onset can predict performance. At first this finding might seem counterintuitive, because it suggests that a boost in attention just before the target actually impairs target detection. Note, however, that the initial increase before target onset on miss trials probably reflects false alarms to distractor letters preceding the target (for a detailed analysis supporting this interpretation, see Figure A4). In other words, the increase in power before target onset on miss trials appears to be a consequence of an increase in attention to non-target letters that look similar to the target (e.g., the letters K and the Y). Presumably drawing attention to these confusable distractor letters occupies attention and prevents detection of the target letter, causing a miss.

Next we determined how well oscillation power dissociated hits from misses using a signal detection ROC analysis. We computed the ROC curves and AUC on the power values of all trials separately for each observer. As indicated by the average AUC for the hit and miss power distributions (for details, see Methods), pupil oscillation power significantly predicted successful target detection before target onset (Figure 3E; *black trace*). The prediction of detection performance was significantly larger than chance at 500 ms before target onset across all observers [0.46 ± 0.08; *t*_(24)_ = 2.37, *p* = 0.026]. Similar to the 2 Hz power values, raw baseline pupil size was also distinct for hits as compared to misses before target onset. To show that PFT has an advantage over the use of raw baseline pupil size, we compared how well each measure explains the probability to hit or miss a target. The analysis of the AUC of the hit and miss baseline pupil distributions shows that raw pupil size also predicted target hits (Figure 3E; *gray trace*). The AUC's for pupil size across observers were significantly larger than chance at 250ms before target onset [0.46 ± 0.08; *t*_(24)_ = 2.56, *p* = 0.017]. However, pupil oscillation power values dissociated hits from misses at ~300 ms earlier than raw pupil size. The raw baseline pupil size, however, dissociated these conditions at 100 ms later than the PFT method. This implies that the PFT method is a more sensitive and earlier predictor of when observers are about to miss a target or not. We further determined whether the power values dissociated between hits and misses after target onset with a higher accuracy than the baseline pupil. Indeed, the peak AUC for power distributions [absolute AUC difference from 0.5 for peak power at 500ms: 0.15 ± 0.10, corresponding to 65% prediction accuracy with 50% being chance) was significantly higher than trough and peak AUC for pupil baseline distributions (difference of trough baseline at 250 ms: 0.09 ± 0.09, corresponding to 59% prediction accuracy; difference of peak baseline at 650 ms: 0.09 ± 0.13; peak power vs. trough baseline: *t*_(24)_ = 3.06, *p* = 0.005; peak power vs. peak baseline: *t*_(24)_ = 3.13, *p* = 0.005]. The difference in power for hit and missed targets at 500 ms was 8 ± 5 units which corresponds to a 38 ± 9% increase in amplitude size (i.e., ~0.08 ± 0.02 mm). In summary, the amplitude of pupil oscillations successfully predicted target detection performance during an attentional tracking task and differentiated better between target misses and hits than raw pupil size.

### General discussion

We successfully probed the allocation and focal strength of attention by frequency–tagging stimuli and simultaneously measuring the flicker-induced pupil oscillations. In the first experiment we verified that the pupil oscillates in response to repetitive onsets and offsets of stimuli up to ~2.5 Hz (Alexandridis and Manner, 1977). In a second experiment, we induced pupil oscillations at around ~2 Hz frequency rates and measured how attention affected the oscillation amplitudes. By conducting a frequency spectrum analysis, we showed that pupil amplitudes were selectively enhanced at the attended stimulus' frequency. Thus, the tagging of multiple stimuli with separate frequencies and the measurement of pupil oscillations successfully indicated which stimulus was attended. In the third experiment we explored the applicability of this PFT method to predict behavioral performance from the pupil oscillations during an attentional tracking task. The pupil amplitudes reflected degrees of attentional allocation that could be used to indicate misses or successful detection of a target. The PFT method is distinct from standard baseline pupil size measurements and also a better indicator of attentional behavior than the much slower arousal-induced pupil dilations. To our knowledge, this study is the first to show that PFT enables the on-line measurement of attentional resources at relatively high spatiotemporal resolutions. Thus, PFT is a new and unprecedentedly powerful tool that extends the limited repertoire of psychophysical and non-invasive neuroscientific methods to study attention.

The question remains how attention modulates pupil oscillations. It is widely known that the iris reflexively regulates the amount of light hitting the retina by changing the pupil's size (Loewenfeld and Lowenstein, 1993), but a few popular publications have introduced the idea that cognitively aroused states and increased mental effort can cause pupil dilations (Kahneman, 1973; Janisse, 1977; Andreassi, 2000; Beatty and Lucero-Wagoner, 2000). Several other discoveries showed that the visual processing and encoding of salient stimulus changes can also cause pupil constrictions (Kohn and Clynes, 1969; Barbur et al., 1992; Naber et al., 2012, 2013; Naber and Nakayama, 2013). Binda et al. (2013) recently found that the pupil constricts if attention is diverted to a bright and salient stimulus. These studies indicate that the amount of attention or processing resources devoted to an event or stimulus affects the amplitude of pupillary responses. Here we show that attention enhances pupil responses in both directions. When attention was diverted to a flickering stimulus, the amplitudes of both the dilation and constriction pupil responses to this stimulus' frequency were enhanced. In addition, we observed an increase in pupil oscillation right after a detected target. The latter effect might due to a boost of attention or the initiation of stimulus encoding, as suggested by models of serial stimulus presentations in attentional blink studies (Chun and Potter, 1995). Alternatively, selective attention may enhance the initial constriction response to the onset of a target (Binda et al., 2013) which is then followed by a dilatory arousal response to the visual detection of the relevant but infrequent target (Hakerem and Sutton, 1966; Friedman et al., 1973). Finally, it is unlikely that the increase in oscillation amplitudes was due to button presses because motor responses result in pupil dilations, not oscillations (e.g., Einhäuser et al., 2010). In sum, these findings suggest that the effects of attention on pupil size are distinct from arousal. Attention enhances pupil responses triggered by a visual event while arousal is more likely to slowly increase baseline pupil size. Hence, our findings extend the contemporary models of arousal as an underlying mechanism for cognitively induced pupil responses and future research may focus on the interaction between selective attention and arousal.

Not many studies have associated transient pupil responses with visual spatial attention. While early work suggested that task-difficulty and effort cause pupil dilation (Kahneman and Beatty, 1966; Pratt, 1970; Libby et al., 1973), only recent work has found that spatial attention may affect the pupil. One study has reported a relation between the spatial spread of attention and pupil size (Daniels et al., 2012). If observers attend objects in the periphery, pupil size is large, whereas pupil size is small when foveal objects are attended. Besides such phasic, low-frequency changes in pupil base-line, the pupil can also change more transiently, for example in response to the onset of a visual stimulus (Barbur et al., 1998; Naber et al., 2012; Wierda et al., 2012). Karatekin et al. (2004) noted a similar distinction in a dual task paradigm with an auditory digit memorization and a reaction time task. They found that baseline pupil size is elevated when subjects perform the two tasks in parallel, but that the pupillary dilations to auditory digits are weakened due to the attentional divergence to the second reaction time task. In line with these studies, we revealed that selective attention modulates transient pupillary responses of observers, independent of other factors such as arousal, effort, and depth of focus. We propose that focal spatial attention enhances pupil oscillations by increasing pupillary response sensitivity to stimulus changes, rather than only increasing baseline pupil size (e.g., Kahneman and Beatty, 1966; Kahneman, 1973). Thus, when a sensory event causes the pupil to either dilate or constrict, attention enhances this response.

Given that attention facilitates the processing or appearance of visual features such as local, *spatial* changes in luminance (e.g., Martínez-Trujillo and Treue, 2002; Williford and Maunsell, 2006; Reynolds and Heeger, 2009), attention may similarly facilitate the neural responses to *temporal* changes in luminance by the on-and-off flicker of stimuli in early sensory brain areas. Indeed, visual structures project to the brain stem nuclei that control pupil size in mammals and birds (e.g., Distler and Hoffmann, 1989; Gamlin and Reiner, 1991; Loewenfeld and Lowenstein, 1993). It is also possible that projections from parietal areas to subcortical targets (Weber and Yin, 1984; Glickstein, 2003) enable attention to boost the neural signal that carries the information to either dilate or constrict the pupil. Further research is, however, necessary to examine whether these attentional enhancements happen at early sensory stages of stimulus processing and/or at later time points when feedback signals progress to drive pupil size.

In addition to raising fundamental questions regarding how attention modifies pupil responses, the present study demonstrates that PFT is a promising test-bed to study the attentional enhancement of visual processing of stimuli in general. For example, future research may focus on the validation of PFT and its relation to SSVEP during the tracking of attention across multiple objects (Müller et al., 2003; Störmer et al., 2013). Other work can focus on the properties of attentional allocation, such as its resolution (He et al., 1996) and biases across the visual field. For example, we find that flickering items in the upper visual field induce stronger pupil responses. An attentional bias for object and shape detection in the upper visual field may account for this (Previc, 1990) and PFT may help to accurately map the spatial extent of such biases. PFT may also be particularly useful in the context of fast spatial shifts in attention due to its relatively high temporal resolution. In addition, PFT may be applied to communicate with Locked-In Syndrome patients (Stoll et al., 2013), diagnose attentional disorders in psychiatry (Graur and Siegle, 2013), or study phenomena such as multiple object tracking (Pylyshyn and Storm, 1988), the attentional blink (Raymond et al., 1992), and inattentional blindness (Mack and Rock, 1998).

## Conclusions

We find that PFT can provide insights to where and how much attention is allocated (or attracted) to visual features. We suggest that the amount of attentional resources devoted to a stimulus onset directly affects the amplitude of the pupil response. A causal link between this form of focal spatial attention and pupillary responses has not been demonstrated before. As a neuroscientific explanation for our findings, we propose that attentional processes innervate the autonomic nervous system, either amplifying contrast sensitivity over time or the neural dynamics driving pupil size. Both pupil dilations and constrictions—as responses to events—are enhanced by attentional resources available at those moments. PFT provides a new method with the potential to decode the dynamics of visual attention and its role in the brain's sensory processes.

### Conflict of interest statement

The authors declare that the research was conducted in the absence of any commercial or financial relationships that could be construed as a potential conflict of interest.

## Figures and Tables

**Table A1:** **Statistical two-sided paired *t*-test comparisons of FFT pupil power between presented and absent frequencies (*n* = 13)**.

**Stimulus frequency (Hz)**	***t*-statistics**	***p*-value**
0.3	28.41	2.248 * 10^−12^
0.7	22.23	4.042 * 10^−11^
1.1	7.81	4.815 * 10^−6^
1.7	8.35	2.424 * 10^−6^
2.3	6.92	1.609 * 10^−5^
3.4	2.73	0.018

**Table A2:** **Statistical two-sided paired *t*-test comparisons of FFT pupil power between target and distracter frequencies (*n* = 15)**.

**Target frequency (Hz)**	***t*-statistics**	***p*-value**
1.50	5.94	3.637 * 10^−5^
1.75	5.13	1.527 * 10^−4^
2.00	5.70	5.469 * 10^−5^
2.25	2.82	0.014

## Appendix

### Experiment 3

#### False alarms

In Experiment 3, observers had to attend a stream of letters that circulated in the periphery and press a button whenever they had seen a target letter “x”. Observers occasionally false alarmed, that is, they reported to have seen a target although no actual target was shown. We here investigated which factors contributed to the occurrence of false alarms. We propose that (1) targets were more likely to be missed directly after false alarms, (2) false alarms were caused by inter-letter confusions, (3) the onset of confusing target–like letters before target onsets induced misses, and (4) false alarms explained the increase in pupil power for misses as reported in Figure 3D. To support the first proposition (i.e., missed targets were preceded by false alarms), we show that the false alarm probability before target onset was higher for missed targets as compared to hit targets (Figure A4A). To support the second proposition (i.e., false alarms were caused by confusing letters), we show that the probability to false alarm depended on the probability to confuse a non-target letter for the target letter “x”. The probability to confuse letters depends on letter similarities (e.g., Bouma, 1971; Loomis, 1982; Gervais et al., 1984). Thus, letters that looked like an “x” had a high probability to cause false alarms whereas other “non-x-like” letters had a low probability to be confused for the target. To get an objective measure of how similar a letter was to the target, we calculated the amount of pixel overlap between the “x” and all other alphabetical letters (Figure A4B). This is a commonly used method to estimate letter confusion probability matrices. Indeed, the amount of overlap between the letters presented in our experiment was roughly in line with confusion probabilities from other studies (e.g., Gervais et al., 1984). As indicated by these overlaps, letters such as “k”, “y”, and “w” have very similar appearances as the target letter “x” (Figure A4C). More important, a false alarm was more likely to be preceded by the onset of letters with high “x-like” confusion probabilities (Figure A4D). To support the third proposition (i.e., the onset of confusing letters before target onset induces misses), we demonstrate that missed targets were preceded by letters with higher confusion probabilities (Figure A4E). In other words, the presentation of “x-like” letters resulted in increased probability of subsequent target misses. Lastly, the fourth proposition (i.e., similar to hits, false alarms also induce increases in pupil power) is supported by the finding that the increase in pupil power before missed targets was larger when letters with high confusion probabilities preceded target onsets (Figure A4F). With these results taken together, we conclude that the false detection of apparent “x-like” non-targets cause a temporary increase in pupil power (see Figure 3D) and a temporary increased probability to miss targets that followed shortly after a false alarm. Thus, false alarms to target-like letters tend to inhibit the detection of subsequent targets and cause increases pupil power as if observers had detected a real target.
